# 

**Published:** 1862-01

**Authors:** 


### INDEX TO VOLUME III.

Page.

Acetate of Potash in Gonorrhœa, 455
Aconite and Nux Vomica, antidotal to each other,............... 254
Agassiz, Honor to,.................... 125
Alcohol, "Is Alcohol Food,"..... 572
American Medical Association, 116, 187, 705
Anæmia, Clinical Lecture on,.... 235
Anæsthesia, Local from Cold,.... 502
Aneurism, Treatment,...........346, 454
Andrews, Prof. E., Clinic in Mercy Hospital,.................. 153
Andrews, Prof. E., Surgeon of Artillery,............................ 249
Andrews, Prof. E., Letters from the War,............342, 479, 597, 669
Artificial Flowers, Green, Dangerous,.................................. 537
Adhesive Strap, Counter-extension Improvement,............... 26
Asthma, New Remedy for,........ 255
Army Assistant-Surgeons,......... 452
Army Medical Officers not Belligerents,.............................. 390
Army Matters, 248, 393, 441, 531, 576, 648
Arsenic and Sesquicarbonate of Ammonia in Ague,................ 375
Artificial Limbs for Soldiers,...... 576
Belladonna in Mammary Abscess, 22
Blane Medals,........................ 255
Blaney, J. V. Z., Surgeon in U. S. Army,.............................. 706
Book and Pamphlet Notices, see pp. 49, 50, 52, 53, 111, 114, 174, 179, 191, 244, 249, 316, 317, 318, 329, 376, 380, 441, 442, 444, 510, 512, 577, 578, 584, 645, 669, 706
Bowel Affections of Children, Clinics on, by Prof. Davis,...459, 538
Cancerous Diseases, Treatment of, 662

Page.

Cantharides Internally,............ 364
Cerebral Congestion, by Dr. Ormsby,.............................. 319
Camp Douglas, Changes in,....... 389
Chicago Medical Society, Meetings of,...........28, 232, 354, 512, 648
Chloroform and Ether, Effects of, &c....................56, 63, 104, 497
Clergyman's Sore Throat,.......... 62
Coffee,..................................... 581
Congenital Malformation,......... 22
Carotid Artery, Ligature of,...... 651
Clinical Cases in the Mercy Hospital,................................446, 654
Country Practitioner,............... 190
Chlorate of Potassa, for Fœtid Breath,............................... 56
Chlorine and Milk, Treatment of Scarlet Fever,...................... 485
Chloride of Lime as an Insecticide, 441
Complex Labor,....................... 13
Deville, Prof. Titus,................. 54
Diarrhœa, Chronic,.................. 449
Drumreicher, C., Assistant-Surgeon, United States Army,..301, 706
Eaton Medical Society, Ohio,.... 700
Eligible Pharmaceutical Formula,.................................. 145
Epilepsy,......................431, 491, 518
Ergot, Medical Uses of,............ 658
Eruptive Fevers, General Pathology of,.............................. 604
Eye-ball, Sympathetic Inflammation of,............................ 557
Explanations,......................... 773
Food in Typhoid Fever,........... 192
Foreign Body in the Air Passages,................................. 229
Gunshot Wounds,.................... 535
Hæmatocele, Uterine and Pelvic, 39, 376, 107
Hairless Men of Australia,........ 456

Page.
Heart, Diseases of,.................... 446
Health of British Army and Navy,.................................... 509
Hollister, Prof. J. H., Valedictory Address,........................ 215
Hemorrhage, Uterine and Pulmonary,............................328, 553
Hospitals, Military,...97, 442, 577, 711
Human Salamander,................. 455
Hydrastis Canadensis, Alkaloids of,........................................ 609
Hopp, William, Trial for Murder, 700, 763
Head, Injuries of,...................16, 418
Illinois State Medical Society, 118, 246, 647
Inversion of Uterus, Spontaneous,......................................... 580
Infusoria, Experiments on Formation of,............................ 578
Iron, Preparations and Effects, 31, 157, 518, 521
Kidneys, Fatty Degeneration of, 405
Labors, Dry, New Treatment of, 47
Leucorrhœa in Pregnant Women, 57
Liver in Cholera Infantum,....... 583
" Excretory Function of, 617, 671, 733
Lind University, Medical Department, 56, 124, 188, 389, 451, 571
Literature and Art................... 329
Letters from the Army,...293, 299, 669
Marriages of Consanguinity, Effects of,.........................338, 374
Masturbation,.......................... 523
McFarland, Dr. And., Letter from, 763
Medical Schools,....................188, 517
" Council of Great Britain, 584
" News,............................ 457
" Societies,.....................54, 393
" Police, Domain of,........ 443
Metropolitan Health Bill of New York,........................................ 247
Milksickness,.......................... 267
New Remedies, Lectures on,....... 302
New Surgical Propositions and Operations,......................20, 60, 61
New York Ophthalmic School and Hospital,....................328, 646
Narcotics,—Belladonna,............ 632
Page.
Nature in the Cure of Disease,... 45
Obituaries,.............125, 189, 249, 577
Obstetric Bag and Instruments,. 563
Oiled Paper in Surgical Dressings,....................................... 440
Opthalmia,................................ 456
Orchitis, Extirpation of Testicle, 414
Ovarian Tumors, &c., 65, 70, 129, 193, 520, 732
Ozone and Miasmata,............... 521
Paracentisis,............................ 372
Paralysis,...........................167, 250
Poisons,.........................455, 456, 631
Phthisis,.................................. 58
Preservation of Animal Food,... 643
Propylamin,............................. 125
Placenta Prævia and Retained, 331, 721
Puerpural Fever,...................... 506
Practice of Medicine, &c., in Modern Times,....................... 728
Pyæmia and Hospital Gangrene, 35
Prize, Cooper's,........................ 644
Quinine in Dropsy after Scarlatina,.................................... 59
Rail Roads, Medical Provision for,......................................... 699
Reproduction of Inferior Maxilla, 58, 127,
Roskotten, Dr., Attack on, and Reply,...............................421, 577
Reports on Important Subjects,.. 88, 119, 126, 192, 290, 381, 393, 395, 438, 471, 545, 587, 706,
Strychnia and Its Uses,...........409, 508
Sponge Pessary,....................... 452
Syphilitic Vaccination and Ecthyma,............................57, 447
Salivary Calculus,.................... 697
Silver Mixture and Nervous Sedatives,.................................. 715
Spermatorrhœa,........................ 713
Splint for the Femur,................ 601
Temperature and Ventilation,... 191
Throat, Diseases of,.........392, 511, 649
Tobacco,................................... 560
Urine and Uremia,.........284, 641, 724
Vaccination in Whooping-Cough, 698
Vaginismus, Dr. Simms on,...355, 390
Varicose Ulcers of the Leg,....... 582
Veratrum Viride,...................... 96

**N. S. DAVIS, M. D., Editor.---FRANK W. REILLY, M. D., Ass't Editor.**

A MONTHLY JOURNAL,

DEVOTED TO THE

**EDUCATIONAL, SCIENTIFIC, AND PRACTICAL INTERESTS**

OF THE

MEDICAL PROFESSION.

The Examiner will be issued during the first week of each month, commenceing with January, 1860. Each number will contain 64 pages of reading matter, the greater part of which will be filled with such contents as will directly aid the practitioner in the daily practical duties of his profession.

To secure this object fully, we shall give, in each number, in addition to ordinary original articles, and selections on practical subjects, a faithful report of many of the more interesting cases presented at the Hospitals and College Cliniques. While aiming, however, to make the Examiner eminently practical, we shall not neglect either the scientific, social, or educational interests of the profession. It will not be the special organ of any one institution, society, or clique ; but its columns will be open for well-written articles from any respectable member of the profession, on all topics legitimately within the domain of medical literature, science and education.

Terms, $2.00 per annum, invariably in advance.

